# From mechanisms to meanings: toward a content-sensitive psychiatry

**DOI:** 10.3389/fpsyt.2026.1742330

**Published:** 2026-03-06

**Authors:** Lucas Ferrer Nappe

**Affiliations:** Short-Stay Psychiatric Inpatient Unit (UHCEP), Hospital Intercultural Kallvu Llanka, Cañete, Chile

**Keywords:** content-sensitive psychiatry, ecological momentary assessment (EMA), meaning, phenomenology, predictive processing, RDoC, subjective experience

## 1 Introduction

Neurobiological advances—from Global Neuronal Workspace (GNW) and Integrated Information Theory (IIT) to predictive processing—have transformed how we model conscious access, integration, and inference. These frameworks primarily explain capacities and mechanisms (how information becomes globally available, is integrated, or is prediction-driven), not what is experienced in a situation nor what it means for a person. Clinically, this gap appears when matched autonomic arousal is thrill on a rollercoaster but dread during trauma recall, or when identical nociceptive input is relief in one frame and suffering in another. Mechanisms are necessary; meanings steer care. This Opinion argues for a content-sensitive psychiatry that treats first-person experience as essential clinical data, in complementarity—not opposition—to neurobiology. We propose a content-sensitive agenda that treats experiential content as structured data, makes context explicit as a constraint on interpretation, and links these variables to biological and computational measures for testable mechanism–content–context coupling.

## 2 Mechanisms are not meanings: working definitions and underdetermination

In this Opinion, we adopt four working definitions to reduce ambiguity. By mechanism we mean the causal physiological or computational processes that generate and shape mental states, including inferential architectures emphasized by predictive processing and related models (1, 2). By content we mean the reportable experiential episode itself (what shows up in experience in a given moment, including its immediate ‘aboutness’). By meaning we mean the person-level significance assigned to that content in light of goals, identity, values, and anticipated consequences (what is at stake). By context we mean the situational, relational, cultural, and temporal constraints that shape which meanings are available or dominant in a given episode.

We treat the content–meaning distinction as pragmatic and clinically operational: the same content can support multiple meanings depending on context.

GNW models conscious access via large-scale ignition and recurrent processing; it is a theory of broadcast/amplification, not semantic content (3). IIT quantifies system integration grounded in phenomenological axioms yet remains a theory of causal structure, not of situated meanings (4). The free energy/predictive processing program explains perception and action as hierarchical inference under uncertainty; it shows how priors and precision shape experience without specifying which meanings a person will adopt in lived contexts (1, 2). Clinically, a noradrenergic profile does not tell us whether a person is frightened, angry, or excited. Constructionist emotion research similarly shows that brains categorize interoceptive change using learned concepts relative to situation, yielding divergent experiences from similar bodily states (5). Even when multivariate neuroimaging can classify broad affective or regulation states above chance (6–8), such signatures indicate that regulation is engaged, not what it means to the person, nor which appraisal organizes behavior.

This distinction also connects to a long-standing issue in consciousness science: mechanistic descriptions can explain enabling conditions and causal organization, yet still leave open questions about experiential character and its interpretation, often discussed under the “hard problem” framing (9). The point here is pragmatic rather than metaphysical: mechanisms are necessary for experience, but meanings guide care.

For these reasons, inferring content from mechanism is underdetermined without context. This is not merely epistemic uncertainty but a structural property: multiple meaning-structures can be compatible with the same physiological configuration unless contextual constraints are specified (5, 10). Physiology may narrow the space of plausible interpretations, but context is required to select among them in a way that is clinically actionable.

## 4 Neuroscience already shows that context reconfigures experience

Placebo/expectancy research demonstrates that appraisals re-weight nociception and alter felt quality. Classic and subsequent fMRI studies show that expectation of analgesia dampens pain-responsive regions and recruits prefrontal/anticipatory control (11, 12); recent work suggests placebo impacts systems for analgesia more than nociception per se (13). Behaviorally, identical inputs can be experienced as relative relief—sometimes pleasant—when embedded in a context of expected worse pain (10). Predictive frameworks generalize this: malleable priors about self and world alter percepts, actions, and the felt sense of states (1, 2, 14). Psychiatry should therefore treat content and meaning—reported labels, appraisals, and identity stakes as a clinical variable, not a narrative add-on.

## 5 Capturing the “missing data type”: content, with rigor

To avoid talking past each other, it is useful to distinguish three limiting tendencies that often appear in clinical reasoning and in discussions about measurement. One tendency prioritizes symptom-count thresholds and standardized severity ratings, which improves reliability but can leave the semantic structure of distress under-specified. A second tendency assumes that increasingly fine-grained biology will, by itself, recover experiential meaning, an expectation that sits uneasily with the underdetermination of content without context (5, 10). A third tendency treats meaning as exclusively narrative—clinically valuable, but difficult to compare longitudinally or to integrate with biological work. Content-sensitive psychiatry rejects none of these outright; it argues that each remains incomplete without structured content variables.

Clinicians already collect fragments of experiential content, usually as free text that is hard to compare longitudinally. Two low-burden strategies can raise rigor. First, structured experiential prompts (chart verbatim; ~5 minutes): What does this state mean to you when it begins? When does the same body feeling mean something different? What prediction about yourself/others seems confirmed in that moment? What would count against that meaning while it is happening? Which actions become possible or impossible when it starts? These elicit situation-bound meanings and belief dynamics that guide behavior.

Operationalization can remain explicit and minimal. Clinically decisive content can be captured as a patient label and a one-sentence meaning statement (“When this state begins, it means X”), complemented by a brief stakes clause and a note on affordances (which actions feel possible or impossible). Context can be recorded with short tags (where, with whom, activity, time-of-day). This converts narrative into variables trackable over time within established ambulatory assessment and experience sampling methods (15, 16).

Second, Ecological Momentary Assessment/Experience Sampling Methods (EMA/ESM) focused on meaning (not only intensity): brief prompts twice daily can sample (a) current bodily label (free text), (b) situational meaning (patient-generated tags), and (c) stakes (what could go wrong/right)?. Reviews and large-sample studies show feasibility and good compliance in mental-health populations (17–19); foundational work in ambulatory assessment details reliability and validity of EMA-derived parameters for emotion dynamics and decision variables (15, 16). Clinically, targets become label flexibility and context–meaning coupling, not only frequency or intensity. Visualizing when identical interoceptive patterns receive different labels across contexts yields content–context maps that can guide psychoeducation and exposure.

## 6 “Five-by-five” implementation (feasible tomorrow)

As a minimal illustration of how these tools can be implemented without increasing clinical burden, five questions in five minutes (record verbatim), a five-minute chart add-on (meaning statement plus counter-label), and a five-minute weekly EMA review (plot label flexibility and context–meaning coupling) can replace unstructured talk, clarify targets, and streamline handovers.

## 7 Phenomenology as method, not abstraction: EASE/EAWE in practice

Phenomenological psychiatry provides structured descriptors of self- and world-experience (e.g., altered mineness, temporality, embodiment, and world-relation) that can be operationalized with semi-structured interviews. The EASE (Examination of Anomalous Self-Experience) shows inter-rater reliability and clinical utility in first-episode psychosis and schizophrenia-spectrum conditions (20), and the EAWE (Examination of Anomalous World Experience) complements EASE by mapping profiles of anomalous world-experience (21). Used pragmatically, these tools convert first-person anomalies into comparable clinical variables that can sharpen formulation and guide targeting.

## 8 Clinical payoffs

If subjective meaning can be measured with rigor, several immediate clinical implications follow. Diagnostics: similar behaviors can instantiate different meaning-structures. Non-suicidal self-injury may enact punitive self-narratives, acute anxiety reduction, or interpersonal signaling; each demands different levers. Treatment selection and timing: mechanism-targeting pharmacotherapy and meaning-targeting psychotherapy work best when guided by which meaning-structure maintains distress (catastrophic appraisal, moral injury, identity threat). Even in medication-first pathways, psychoeducation on construction and prediction can change how arousal is interpreted (5, 11–13). Relapse prevention via re-labelling: practiced counter-labels (e.g., “this arousal is mobilization, not danger”) can redirect behavior with the same physiology; expectancy/placebo literatures show measurable experiential and neural effects (11–13). Family and team alignment: family involvement improves acute care outcomes but is rarely standardized at the content level (22). A one-line “meaning statement” (e.g., “Panic = impending humiliation at work”) plus a negotiated counter-label aligns relatives and staff and clarifies crisis plans.

## 9 Anticipating objections (with evidence)

Any proposal that treats experience as data invites predictable objections. “Subjectivity cannot be measured.” It already is, routinely, via standardized self-report instruments and visual analog scales. The more precise concern is that subjective experience is often measured in low-resolution ways that omit context and semantics. EMA/ESM adds density and context with good compliance and validated parameters across mental-health populations (15–19).

“Mechanisms will subsume meaning.” Multivariate approaches and neural signatures can classify broad affective or regulatory states above chance (6–8), but they under-specify semantics; content depends on learned concepts and appraisals that are not uniquely recoverable from physiology absent contextual constraints (5). The pragmatic path is complementary: refine mechanistic models while measuring meaning directly.

“This will slow clinics.” The proposed elements are brief and templated; in practice, a structured segment can replace unstructured conversation, clarify targets, and improve handovers. The goal is not more data, but better data: explicit meaning statements and context tags that reduce ambiguity in formulation and planning.

## 10 Integrating—not opposing—biology: a research agenda

Content-sensitive psychiatry strengthens biological research by adding testable variables. Here, coupling denotes a reproducible relationship between structured content variables, contextual features, and biological or computational measures, such that changes in meaning statements or appraisals covary with measurable shifts in physiological dynamics or model parameters.

Outcomes can include label flexibility, appraisal change, and context–meaning coupling alongside symptom scales. Tools such as structured prompts, EMA tags, and semi-structured phenomenology provide the measurement layer by turning content into analyzable variables rather than free text (15, 16). Context tags provide the constraint layer that reduces underdetermination and allows within-person comparisons. Mechanistic and computational frameworks then supply candidate parameters for integration, including (but not limited to) precision weighting in predictive processing and physiological proxies such as HRV and pupilometry (1, 2, 14).

Two tractable tests follow. First, mechanism–content–context coupling can be examined by pairing EMA-derived meaning variables with mechanistic proxies and computational parameters to test whether within-person meaning shifts predict changes in biological dynamics under comparable contexts, consistent with predictive accounts of inference and precision (1, 2, 14). Second, treatment mediation can be tested in SSRI and psychotherapy trials by assessing whether changes in meaning statements mediate symptom gains, and whether mediation differs by mechanism- versus meaning-targeting interventions.

## 11 Discussion

Psychiatry excels when it integrates how brains enable experience with what the experience concretely is for a person in context. Mechanistic theories specify conditions for access, integration, and inference; they do not resolve the semantic/pragmatic content that directs action and suffering. A small shift—treating experience as structured data; capturing meanings with EMA and phenomenological tools; aligning teams around explicit meaning statements—can improve diagnostic precision, treatment selection, and relapse prevention while generating new, biologically testable questions at the mechanism–content interface. Beyond the brain, subjective experience remains psychiatry’s most specific signal.
